# Hepatocellular Carcinoma Presenting with Obstructive Jaundice during Pregnancy

**DOI:** 10.1155/2014/502061

**Published:** 2014-08-05

**Authors:** Huan-wei Chen, Feng-jie Wang, Jie-yuan Li, Eric C. H. Lai, Wan Yee Lau

**Affiliations:** ^1^Department of Liver Surgery, The First People's Hospital of Foshan, Foshan, Guangdong 528000, China; ^2^Department of Hepatobiliary Surgery, The First People's Hospital of Foshan, Foshan, Guangdong 528000, China; ^3^Faculty of Medicine, The Chinese University of Hong Kong, Shatin, New Territories, Hong Kong

## Abstract

*Introduction*. Both hepatocellular carcinoma (HCC) presenting during pregnancy and HCC presenting with obstructive jaundice due to a tumor cast in the biliary tract are very rare. The management of these patients remains challenging. *Presentation of Case*. A 23-year-old lady presented with obstructive jaundice at 38 weeks of gestation. Investigations showed HCC with a biliary tumor thrombus. She received percutaneous transhepatic biliary drainage (PTBD) and caesarean section. Right hepatectomy, extrahepatic bile duct resection, and left hepaticojejunostomy were carried out when the jaundice improved. The postoperative course was uneventful. She was discharged home on postoperative day 10. Histopathology showed HCC with a tumor thrombus in the bile duct. The surgical margins were clear. One year after surgery, the mother was disease-free and the baby was well. *Conclusion*. With proper management, curative treatment is possible in a pregnant patient who presented with obstructive jaundice due to a biliary tumor thrombus from HCC.

## 1. Introduction

Although the worldwide annual incidence of hepatocellular carcinoma (HCC) in women is 5.5/100,000–8/100,000 [1–3], HCC during pregnancy is so rare that less than 50 cases have been reported. The rarity of HCC presenting during pregnancy is due to a combination of three factors: male predominance of HCC, rarity of HCC at reproductive age, and decreased fertility in women with advanced cirrhosis [4]. HCC during pregnancy is also believed to have a poorer prognosis than HCC in nonpregnant women [5–7], with 1- and 3-year overall survival rates of 29.5% and 13.6%, respectively [7]. Furthermore, in the majority of cases, pregnancy was terminated when cancer was diagnosed [4–7].

HCC presenting as painless, progressive obstructive jaundice secondary to a biliary tumor thrombus is rare. It is important to diagnose the underlying cause of jaundice in these patients as this affects the management [8–15].

We herein report a pregnant lady who presented with obstructive jaundice due to a biliary tumor thrombus from HCC.

## 2. Presentation of Case

**Figure 1:**
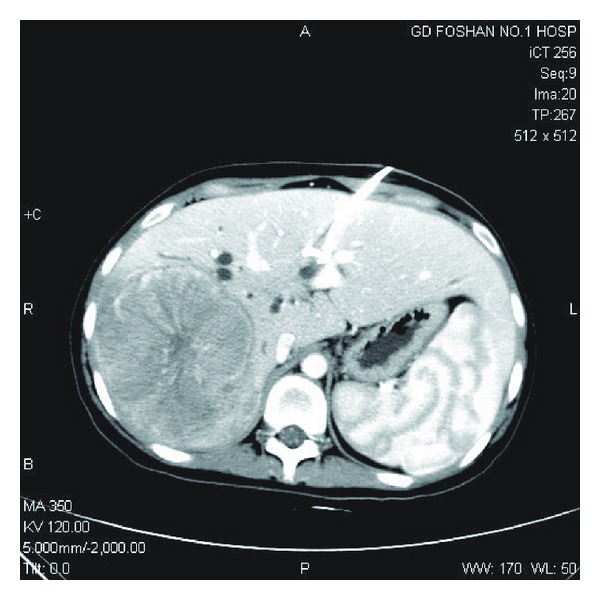
Computerized tomography (CT) angiography showed a HCC at the right posterior sector accompanied by adjacent small satellite foci.

**Figure 2:**
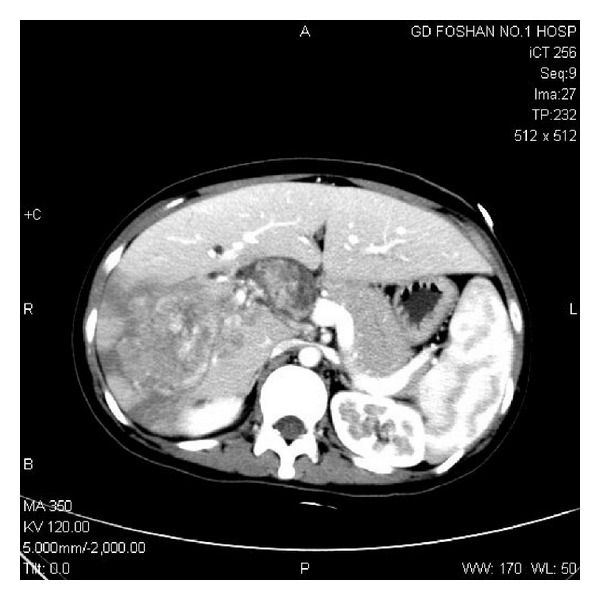
Biliary tumor thrombus at the right posterior sectoral duct, common hepatic duct, and common bile duct.

**Figure 3:**
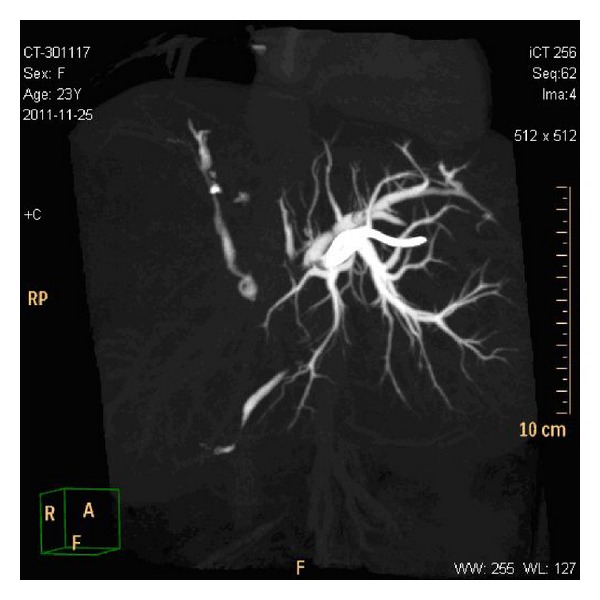
CT cholangiography showed filling defects in the right posterior sectoral duct, common hepatic duct, and CBD.

**Figure 4:**
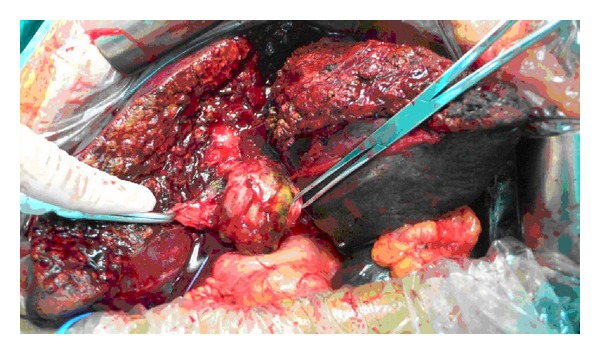
Tumor thrombus in the right posterior sectoral ducts, the common hepatic duct, and the common bile duct.

**Figure 5:**
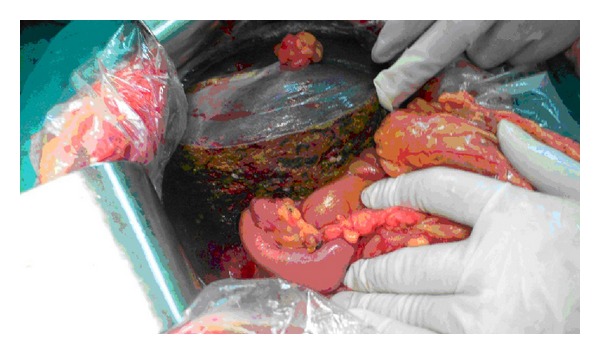
Right hepatectomy, bile duct resection, and left hepaticojejunostomy.

**Figure 6:**
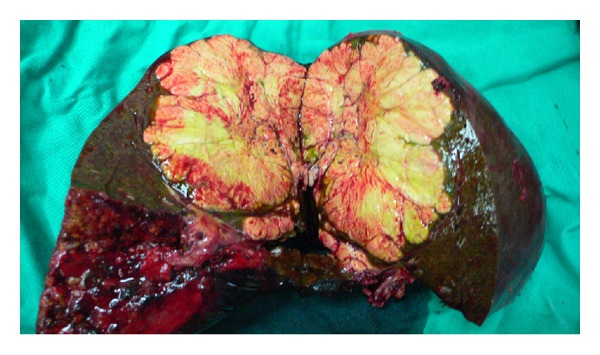
Specimen of the resected liver.

A 23-year-old female, 38 weeks and 3 days pregnant, was admitted to our hospital with a 3-week history of painless, progressive jaundice. Laboratory results showed hepatitis B surface antigen (HbsAg)(+), antibody hepatitis B e-antigen (Anti-Hbe)(+), hepatitis B core antibody (Anti-Hbc)(+), total, direct, and indirect bilirubin levels 183.2 umol/L, 112.1 umol/L, and 71.1 umol/L, respectively, and alpha-fetoprotein (AFP) level 23726 ng/mL. Both ultrasound (USG) and magnetic resonance imaging (MRI) showed a tumor at the right posterior sector of the liver, 114 mm × 108 mm, with adjacent satellite tumors. A tumor thrombus was seen in the right posterior sectorial duct, extending through the right hepatic duct into the common bile duct. The liver was cirrhotic. The diagnosis was HCC with a biliary tumor thrombus. Caesarean section was carried out and a normal male infant with body weight of 3.1 kg was delivered. The patient's jaundice increased and she received percutaneous transhepatic biliary drainage (PTBD) 2 weeks after delivery. The jaundice then improved gradually. At 38 days postpartum, computed tomography (CT) was repeated (Figures 1, 2, and 3). Her liver function was as follows: total bilirubin, 224.2 umol/L; albumin, 41.6 g/L; alanine transaminase (ALT), 49 IU/L; aspartate aminotransferase (AST), 69 IU/L; alkaline phosphatase (ALP), 258 IU/L; *γ*-glutamyl transpeptidase (GGT), 249 IU/L; and preoperative indocyanine green retention rate at 15 min (ICG-R15), 16.8%. She underwent laparotomy, right hepatectomy, extrahepatic bile duct resection, and left hepaticojejunostomy. Liver parenchymal transection was performed with an ultrasonic dissector and coagulative diathermy under vascular inflow control. At operation, there was a HCC in the right posterior sector of the liver, with multiple adjacent small satellite tumors. A tumor thrombus was found in the posterior and anterior sectorial ducts, the common hepatic duct, and the common bile duct (Figures 4, 5 and 6). The intraoperative blood loss was 1300 mL. The operating time was 260 mins. The postoperative course was uneventful. She was discharged from our hospital on postoperative day 10. Histopathology showed a HCC with multiple satellite nodules and a tumor thrombus in the bile duct. The surgical resection margins were clear. One year after surgery, the patient remained disease-free. Both the mother and the baby were well.

## 3. Discussion

HCC presenting during pregnancy is extremely rare. Thus, the experience in its management and in its outcome is not clear. Pregnancy may have an adverse effect on the prognosis of hormonal dependent tumors, although this is still a subject of controversy for HCC. One explanation for the poorer prognosis for HCC points to the elevated levels of sex hormones [16]. In our patient, the bile duct involvement of HCC added to the complexity in the management. For any patient diagnosed with a malignant neoplasm in pregnancy, there are both the mother and the fetus to be considered. For patients presenting in early pregnancy, therapeutic abortion should be offered in order to start anticancer treatment early. For patients presenting in late pregnancy, a thorough discussion with the patient on the management plan and a multidisciplinary management approach among surgeons, obstetricians, and radiologists are paramount. In our patient we were able to offer a treatment with curative intent to the mother and delivered a healthy baby.

The most common presentation of HCC is right upper quadrant discomfort or pain. Jaundice occurs in 5–44% of patients [8–15]. Jaundice is an important clinical presentation in patients with HCC as different aetiological causes of jaundice in HCC determine the therapeutic approach and the prognosis. Based on the underlying pathophysiology, jaundice in HCC can be classified into two types: the hepatocellular type and the icteric type (or the cholestatic type). In patients with the hepatocellular type, treatable causes, such as reactivation of underlying viral hepatitis or alcoholic or drug-induced hepatitis, need to be excluded. If the jaundice is due to hepatic parenchymal insufficiency and without a reversible cause, supportive medical treatment is given. These patients have a very dismal prognosis. The reported incidence of the icteric type of HCC varies from 0.5 to 13% of all patients with HCC [15]. The icteric type of HCC can be attributed to one of the following mechanisms: (i) a tumour may erode into a branch of the biliary tree and grows distally until it fills up the entire extrahepatic biliary tree to form a biliary tumor cast in the extrahepatic bile ducts; (ii) a necrotic free-floating fragment of tumor may separate from the biliary tumor cast and migrate distally to obstruct the common bile duct; fragments of tumor in the bile duct, as described by Edmonson and Steiner, are usually fragile, fleshy, and grey-white and have the appearance of chicken fat; and (iii) sometimes, bleeding from the biliary tumor cast may partially or completely fill up the biliary tree with blood clots that obstruct the biliary system. With increasing recognition of the icteric type of HCC, a classification with therapeutic implication is needed. Lai and Lau classified the icteric type of HCC into the extrahepatic and the intrahepatic types [14, 15]. This classification has important therapeutic and prognostic value as patients with extrahepatic biliary obstruction secondary to HCC have a higher curative resection rate, which results in a significantly improved survival rate when compared with those patients with intrahepatic biliary obstruction. Lai and Lau also further refined the classification of the icteric type of HCC based on cholangiographic appearances. Type 1 obstruction is due to intraluminal biliary obstruction caused by either a biliary tumor thrombus or a free-floating tumor fragment. The biliary tumor thrombus gives a cholangiographic intraluminal filling defect appearance that resembles a cork in the neck of a bottle. Lai and Lau termed this radiologic sign the “cork sign.” The cholangiographic imaging features of intraluminal tumor fragments are similar to those seen in choledocholithiasis, but the edges of the filling defects secondary to the tumor fragments are irregular and less well defined than those of stones. Type 2 obstruction is due to haemobilia. The haemobilia gives rise to cholangiographic features of fluffy intraluminal filling defects, which obscure the underlying intraluminal tumor. Another cholangiogram should be carried out after the haemobilia has settled to clarify the actual extent of the intraluminal tumor. Type 3 obstruction is due to extraluminal biliary obstruction. Tumor invasion and/or encasement of the intrahepatic branches of the hepatic ducts give rise to localized strictures with proximal ductal dilatation intrahepatically. The presence of malignant porta hepatis lymph nodes can compress the common hepatic or bile duct leading to extrahepatic biliary obstruction. This classification also bears important therapeutic implications as a significant proportion of patients with the icteric types 1 and 2 HCC have resectable tumors, whereas patients with the icteric type 3 HCC have unresectable diseases with a bad prognosis. Case-controlled studies showed that the long-term survival of patients after curative liver resection of operable HCC with biliary tumor thrombus when compared with those without biliary involvement is similar. Currently, there is still controversy as to whether biliary tumor thrombectomy should be carried out through a choledochotomy instead of carrying out en bloc resection of the extrahepatic duct followed by biliary enteric anastomosis. Based on the current evidence, Lai and Lau proposed that if the biliary tumor thrombus does not infiltrate into the bile duct wall, thrombectomy through a choledochotomy is a viable option for cure. In patients with free-floating intraductal tumor fragments causing biliary obstruction, the curative treatment of choice is to remove the tumor fragments through a choledochotomy followed by resection of the liver segments containing the tumor. Our patient belonged to the icteric type 1 with tumor thrombus extending from intrahepatic duct to extrahepatic duct. She remained disease-free at one year after surgery.

## 4. Conclusion

With an adequate preoperative assessment and management strategy, curative treatment is possible in a patient with extensive HCC during pregnancy.
